# Microstructures and Properties of AlMgTi-Based Metal-Intermetallic Laminate Composites by Dual-Steps Vacuum Hot Pressing

**DOI:** 10.3390/ma13183932

**Published:** 2020-09-05

**Authors:** Linggang Meng, Bingwen Zhou, Bin Ya, Dong Jing, Yingxi Jiang, Danning Zhang, Xingguo Zhang

**Affiliations:** School of Materials Science and Engineering, Dalian University of Technology, Dalian 116024, China; menglg@dlut.edu.cn (L.M.); zbw@dlut.edu.cn (B.Z.); yabin@dlut.edu.cn (B.Y.); jdkuaidi@126.com (D.J.); jyxjob@foxmail.com (Y.J.); Zhangdn958@163.com (D.Z.)

**Keywords:** AlMgTi-based MIL composites, dual-steps vacuum hot pressing, phase composition, micro hardness, bending strength

## Abstract

AlMgTi-based metal–intermetallic laminated composites were successfully fabricated through an innovative dual-step vacuum hot pressing. First, this study prepares the AlTi-based laminated composites by vacuum hot pressing at 650 °C. Then, the researchers place the Mg-Al-1Zn (AZ31) magnesium alloy between the prepared AlTi-based laminated composites at 430 °C for hot pressing. This study investigates the microstructure, phase composition, and microhardness distribution across interfaces of the intermetallics and metal. A multilayer phase (Mg_17_Al_12_, Al_3_Mg_2_, and transition layers) structure can be found from the diffusion layers between Al and AZ31. The microhardness of the material presents a wavy distribution in the direction perpendicular to the layers; the maximum can be up to 600.0 HV0.2 with a minimum of 28.7 HV0.2 The microhardness gradient of an AlMgTi-based composite is smoother due to the different microhardness of the layers, and reduces the interface stress concentration. The bending strength of AlMgTi-based composites can reach 265 MPa, and the specific strength is 105 × 10^3^ Nm/kg, higher than AlTi-based composites.

## 1. Introduction

The metal–intermetallic laminated (MIL) composites have a similar structure to the shell pearl layer. When the material is subjected to bending or impact, the interfaces of metal and intermetallic not only cause the crack propagation path to extend, but also change the deflection direction of the crack and consume energy during the expansion process of cracks [1,2,3]. Concurrently, when the entire material is deformed, the ductile phase will reduce the stress intensity factor at the crack tip and increase the crack propagation resistance through plastic deformation. The MIL composites are the alternate stacking of intermetallic compound layers with high strength and hardness and metal layers with substantial toughness, thus the materials have high strength and hardness, while retaining their toughness [4,5]. The lower density, higher specific strength and modules of the MIL composites make them an attractive material for protection structural applications, such as protective materials of high-speed collision in airspace or ballistic armor for tanks [6,7].

Scholars have widely studied many MIL composite systems, such as Ti/Al [8,9,10,11,12], Al/Fe [13,14], Cu/Al [15], Ni/Al [16] Al/Mg [17,18,19], and Al/Mg/Ti [20]. Among them, the research on AlTi-based composites is relatively mature. The intermetallic compound Al_3_Ti has high strength and high hardness, but its toughness and plasticity are poor. However, AlTi-based MIL composites have exceptional mechanical properties because of their layered system. Even a thin Ti layer can significantly improve the toughness of the composites. MIL composites offer improved mechanical properties; the reason for this is that its multilayer structure has a unique resistance to crack growth and special failure form, such as crack bridging, crack deflection, and crack deflection. However, the AlTi-based composites have only a single intermetallic compound layer (Al_3_Ti); the brittle intermetallic compound Al_3_Ti limits the influence of the interface effect [4,5,21].

In this paper, to increase the strength and toughness of the MIL composites through the interface effect, we prepared a ternary MIL composites by the hot-pressing method according to the characteristics of the AlTi-based and the light AlMg-based MIL composites with multi-phase diffusion layers. Novel AlMgTi-based MIL composites can improve the lightweight performance, obtain the multi-layer intermetallic compound structure, and optimize its bending performance by the interface effect. Given that the reaction temperatures of Al/Mg and Al/Ti differ, preparing AlMgTi-based MIL composites by an innovative dual-steps vacuum hot-pressing is necessary.

## 2. Materials and Methods

The AlMgTi-based MIL composite was designed and prepared, and AlTi-based MIL composite as the comparison experiment. The materials used in this test were annealed Ti foil (Qinghe Metal materials co., Ltd, Xingtai, China, similarly hereinafter) with purity of 99.8% and thickness of 0.5 mm, Al foil with purity of 98.39% and thickness of 0.5 mm, and AZ31 magnesium alloy metal foil with thickness of 0.5 mm. Table 1 presents the chemical composition of foil raw material.

**Figure 1:**
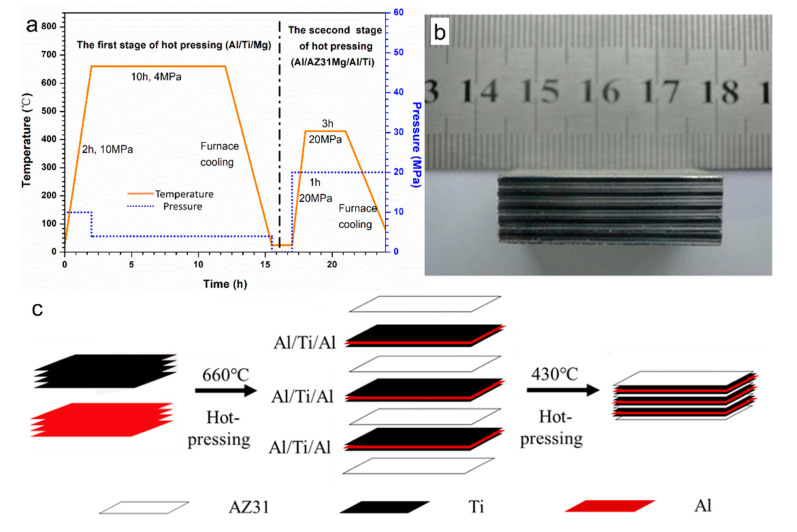
The preparation process of AlMgTi-based metal–intermetallic laminated (MIL) composites: (**a**) Step hot pressing process; (**b**) AlMgTi-based micro laminated material sample; (**c**) Schematic diagram of preparation process.

The researchers first pretreated the surfaces of the raw materials. The procedures: cutting the metal foil to a predetermined size, polishing the surface, washing with water, then ultrasonic cleaning with alcohol, and drying. The vacuum hot-pressing process of AlMgTi-based composites is divided into two steps. In the first step, the best temperature of Al/Ti diffusion by hot-pressing is near the melting point of Al [9], at this temperature, Al and Ti elements have a higher mutual diffusion rate, which can form a certain thickness of diffusion layer at a certain time. AlTi-based MIL composites prepared by 0.5-mm Ti foil and 0.2-mm Ti foil are obtained in this step. The eutectic temperature of Al and Mg is 437 °C; if the eutectic temperature is exceeded, the intermetallic compound formed by diffusion reaction will be remelted, to ensure the good metallurgical diffusion combination of the prepared materials for AlMgTi-based samples. The reaction temperature in the second step is below 437 °C. Figure 1a,c shows the preparation process and schematic diagram. In the first step, the pretreated Al and Ti foil are stacked into three layers of Al/Ti/Al, which reacted 660 °C and 4 MPa in a hot-pressing device. After 10 h, a uniform diffusion layer is constructed between Al and Ti; in the second step, the pretreated AZ31 magnesium foil is placed between the three layers of Al/Ti/Al. Then, the AlMgTi-based MIL composites are hot-pressed for 3 h under a vacuum condition of 430 °C and 20 MPa. Figure 1b is the sample picture of the AlMgTi-based composites.

The Al/Mg/Ti MIL composites samples were mechanically polished with abrasive papers. The morphology and element distribution of the diffusion layers were characterized by optical e (OM, Leica MEF-3, Leica Microsystems, Wetzlar, Germany), scanning electron (SEM, SUPARR 55, Carl Zeiss AG, Jena, Germany) and confocal laser scanning microscopes (CLSM, OLS4000, Olympus Corporation, Tokyo, Japan). The phases were identified by the X-ray diffractometry (XRD, Empyrean, PANalytical B.V., Almere, Netherland) with Cu Kα radiation. The distribution of microhardness perpendicular to the interface was measured by micro Vickers hardness tester (HV-1D, Shanghai Biaoyu Precision Instrument Co., Ltd., Shanghai, China) with 1.961 N of load value (HV0.2). The three-point bending test on a CSS-44200 universal testing machine at room temperature, and the crosshead movement speed was 2 mm/min during testing.

## 3. Results and Discussion

### 3.1. Microstructure

**Figure 2:**
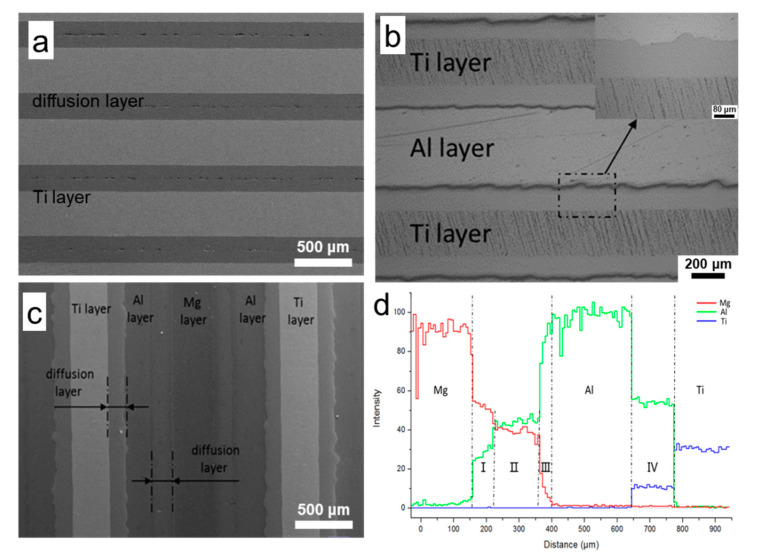
Interface morphology and element distribution of AlTi-based and AlMgTi-based MIL composites: (**a**) The secondary electron SEM image of AlTi-based MIL composites; (**b**) The OM image of Al/Ti/Al prepared in the first stage of the reaction; (**c**) The secondary electron SEM image of AlMgTi-based MIL composite prepared by dual-steps hot pressing process; (**d**) The element distribution lines in the area shown in the red line segment in figure (c).

Figure 2 shows the interface morphology and element distribution of AlMgTi-based and AlTi-based MIL composites. During the preparation of AlTi-based MIL composites, Al layers were completely consumed, and the typical AlTi-based MIL composites according to reference 5 were obtained, as shown in Figure 2a. During the preparation of the AlMgTi-based MIL composites, the microstructure of the three layers of Al/Ti/Al prepared in the first step is shown in Figure 2b, in which the light layer is pure Al layer and the dark layer is the Ti layer. The dense diffusion layers without cracks or pores were formed between Al and Ti layers. The interface between the diffusion layer and Ti is a straight line, while that between the diffusion layer and Al is a tongue-like protuberance. The formation of this morphology is mainly due to the rapid diffusion of the Al element in one direction, and the growth of the intermetallic layer in this direction is significantly faster than that in the other direction [4]. In this stage, Al layers remain in the subsequent reaction between Al and Mg layers during the second steps. Figure 2c shows the microstructure of AlMgTi-based MIL composites prepared through Dual-Step hot-pressing. As shown in this figure, the remaining initial metal layer after reaction is marked, and a diffusion layer exists between the initial metal layers. In the first stage of the reaction, the thickness of the Al-Ti diffusion layer is 150 μm. In the subsequent reaction, although the pressure is sizeable, the diffusion rate of elements is very slow due to the lower reaction temperature, and the slower diffusion makes the thickness of the Al-Ti diffusion layer basically unchanged. The thickness of the diffusion layer formed at the interface of Al-Mg is 190 μm, which indicates that a significant mutual diffusion rate exists between Al and Mg in the reaction near the eutectic temperature, hence a certain thickness of diffusion layer can be constructed in a short time.

**Figure 3:**
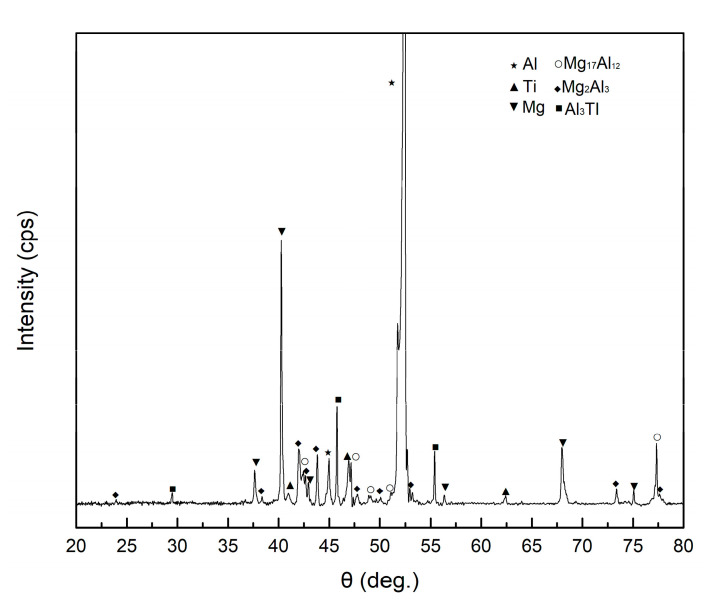
XRD patterns of a cross-section of AlMgTi-based MIL composite.

The phase composition of the diffusion layers in the AlMgTi-based MIL composites were identified by EDS and XRD. Figure 2d shows the EDS line scan analysis results from the AZ31 layer to the Ti layer along the red line in Figure 2c. The original metal layer can be clearly distinguished from the EDS results, but the change in element concentration in the diffusion layers is not a single trend. The concentration change in Mg and Al elements in the diffusion layer between AZ31 and Al can be roughly divided into three different regions, as shown in Figure 2d: near Zone I of AZ31 layer, the content of Mg element is greater, but the concentration change in Al and Mg elements is smaller, and the concentration ratio is fixed; Zone II is the middle layer of the diffusion layer, where the content of Al is greater, but Al and Mg components are uniform; while Zone III is near the Al layer, where the content of Al element rises rapidly. The content of Mg decreased to 0 with the increase in the Al content. In the diffusion layer between Al and Ti, the contents of Al and Ti are fixed. According to the binary phase diagrams of Al-Ti and Al-Mg, the atomic ratios of elements in Regions I, II, and IV are fixed, indicating that they are an intermetallic compound layer, while region III is a solid solution layer of Mg. The XRD result shown in Figure 3 shows that three intermetallic compounds are present in the AlMgTi-based MIL composites: Mg_2_Al_3_, Mg_17_Al_12_, and Al_3_Ti. Therefore, according to the change in element concentration in Figure 2d, Zone I is Mg_17_Al_12_, Zone II is Mg_2_Al_3_, and Zone IV is Al_3_Ti.

The AlMgTi-based MIL composites obtained by the dual-step hot-pressing method have a multi-phase layered structure. Mg_17_Al_12_ layers are present near the Mg layer between Al and AZ31 layer, Mg_2_Al_3_ layers in the middle layer, and composition transition layers near Al layer. However, only one phase is the Al_3_Ti phase between Al and Ti.

### 3.2. Micro-Hardness Distribution

**Figure 4:**
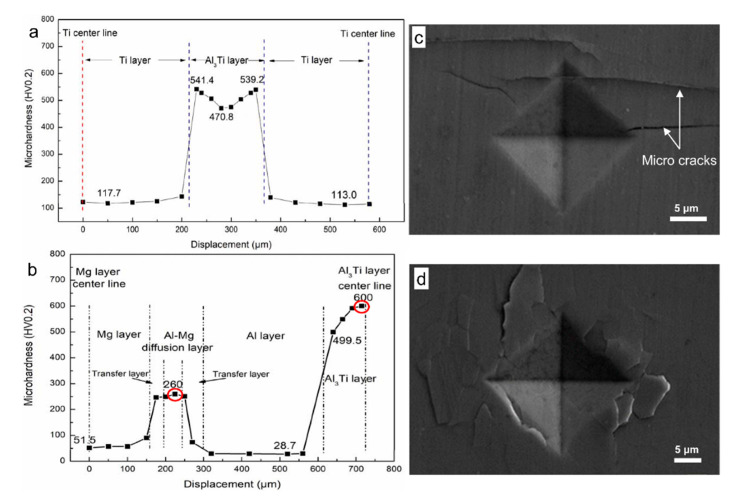
The microhardness distribution and indentations: (**a**) Microhardness distribution at the interface of AlTi-based MIL composite; (**b**) Microhardness distribution at the interface of AlMgTi-based MIL composite; (**c**) Microhardness indentation of Al/Ti diffusion layer; (**d**) Microhardness indentation of Al/Mg diffusion layer.

Figure 4 shows the micro-hardness distribution across the entire reactive region of AlTi-based and AlMgTi-based samples. In the AlTi-based sample, the hardness curve presents a significant fluctuation due to the single Al_3_Ti layers, as shown in Figure 4a. Figure 4b shows that the micro-hardness distribution across the entire reactive region of the AlMgTi-based sample, and the curve presents the more varied hardness gradient due to the different intermetallic and metal layers. The hardness of intermetallic layers are 499.5 to 600.0 HV0.2 (Al_3_Ti layer) and 260.0 to 73.5 HV0.2 (from Al_3_Mg_2_ to Al_12_Mg_17_ layer), and the hardness of metal layers are 113.0 to 143.2 HV0.2 (Ti layer, in Figure 4a), 51.5 to 57.4 HV0.2 (AZ31 layer) and 28.7 to 30.2 HV0.2 (Al layer), which is consistent with the design goal of MIL composites that combine the high strength and stiffness of the intermetallic phases with the high toughness and ductility of the residual metals. However, because the intermetallic compounds are brittle, Figure 4c,d displays that cracks form at the micro-hardness indentations of red areas in Figure 4b, and Al_3_Ti, forming penetrating cracks easily. Moreover, the abrupt change in hardness and stiffness across the metal/intermetallic interface can lead to the stress concentrations causing delamination in the multilayer systems, such as AlTi-based MIL composites [5]. However, the transfer layer between Al layer and Al_3_Mg_2_ smoothens the change in hardness value curve. Moreover, the change range of the hardness value of Al_12_Mg_17_ layer and the Mg layer is small, which effectively reduces stress concentration. Ultimately, Figure 4b exhibits micro-hardness along the direction of the interface perpendicular to the layer, and the layer is loop distribution with an alternate period. According to the toughening mechanism of the MIL composites, along the direction of the interface perpendicular to the layer, this structure can considerably impede the expansion of cracks and improve the composites’ toughness.

### 3.3. Three Point Bending Properties of AlTi-Based and AlMgTi-Based MIL Composite

**Figure 5:**
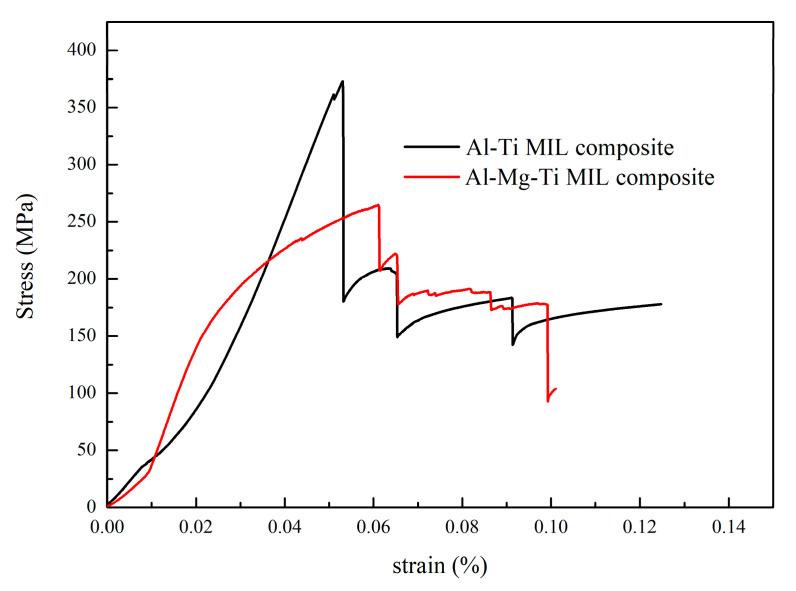
The stress–strain curves of AlTi-based and AlMgTi-based MIL composites.

Figure 5 shows the stress–strain curve of the bending test of the AlTi-based and AlMgTi-based samples. As shown in the figure, with the increase in strain, the stress first increases and then decreases in the AlTi-based and AlMgTi-based samples, which is related to the structural characteristics of the combination of strength and toughness of the MIL composite. Compared with AlTi-based samples, AlMgTi-based samples present more descending tiny steps. The reason for the failure form is that the AlMgTi-based MIL composites are formed by alternately stacking a tough layer composed of the base metal and a strong layer composed of the intermetallic compound. This structure hinders the crack growth. The bending strength of the composite is 265 MPa. Particularly because the density of the AlMgTi-based MIL composite is only 2.51 g/cm^2^, the specific strength of the AlMgTi-based MIL composite is 105 × 10^3^ Nm/kg, higher than the 95 × 10^3^ Nm/kg of the AlTi-based MIL composite, as shown in Table 2.

**Figure 6:**
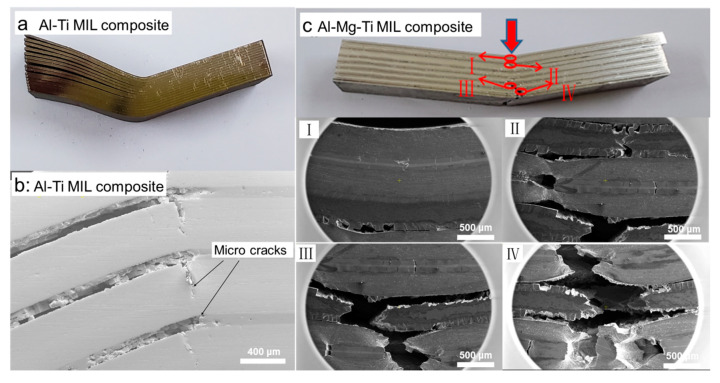
Crack morphology of AlTi-based and AlMgTi-based bending samples: (**a**) The macro morphology of AlTi-based bending sample; (**b**) The micro morphology of AlTi-based bending sample; (**c**) The macro morphology of AlMgTi-based bending sample and four kinds of typical area.

**Figure 7:**
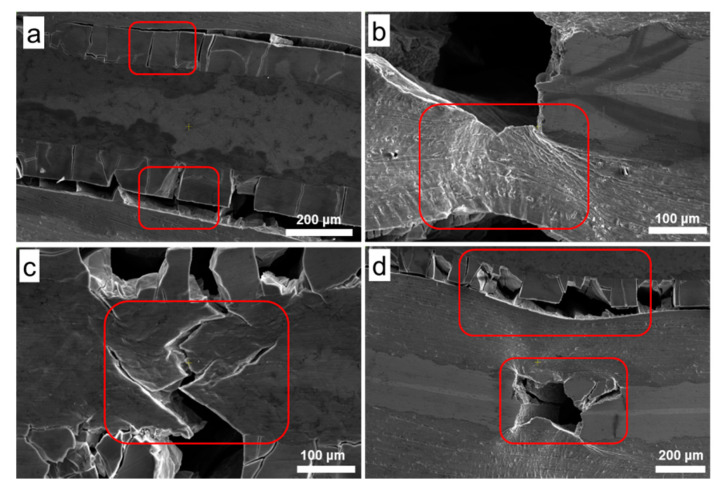
Crack morphology of four kinds of typical areas of AlMgTi-based bending sample: (**a**) Aluminum-magnesium diffusion layer; (**b**) Aluminum metal layer (**c**) Magnesium metal layer (**d**) Aluminum-titanium diffusion layer.

To explore the fracture mechanism of AlMgTi-based samples in the bending test and understand the crack propagation status in each metal layer, the cracks of the bending sample were observed, as shown in Figure 6 and Figure 7. In the AlTi-based samples, the failure modes are the main cracks between the layers, as shown in Figure 6a,b. Figure 6c shows the macro crack morphology and four kinds of typical area of the AlMgTi-based samples. Figure 7 shows the crack scanning electron microscope images of the Al-Mg diffusion, the aluminum metal, the magnesium metal, and the Al-Ti diffusion layers, respectively.

Figure 6c reveals that the crack source of the specimen is formed on the opposite side of the force application direction and propagates to the force application place, and did not present the crack between the layers. Figure 7a,d displays that the direction of the cracks between the diffusion layers are all perpendicular to the direction of the metal layer, and have more cracks. Simultaneously, the Al-Mg diffusion layer shows the more vertical cracks than the Al-Ti diffusion layer. The diffusion layer is a brittle phase, and a large number of cracks are formed under the action of small force, while the diffusion layer of aluminum and magnesium shows higher brittleness. Numerous holes exist in many positions between the metal diffusion layers. The reason is that when a large number of vertical cracks perpendicular to the metal layer are formed, the secondary transverse cracks connect the vertical cracks. Under the action of the force, the specimen deforms, and the metal diffusion layer becomes granular material, and then peels off, resulting in the formation of the above holes. Another phenomenon is that the interlayer separation of Al-Mg diffusion layer often occurs near the Al side, indicating that the interface bonding strength near the Al side is weaker.

Another obvious difference that can be found through Figure 7a,c is that the cracks at the metal diffusion layers are vertical, while the cracks at the metal layers are inclined to expand, with a growth direction of 45° or 135°. Concurrently, the main cracks of the base metal layer are accompanied by many branch cracks, which will stop extending for a certain distance in the ductile layers, and the crack passivation phenomenon will occur at the crack tip. The above situation is due to the brittleness of the diffusion layers and ductile fracture of the aluminum layers. The increase in the crack growth path in the ductile layers consumes more energy, and makes the fracture difficult to occur, enhances the bending strength of the material, and forms more descending tiny steps in the stress–strain curve as shown in Figure 5. In Figure 7b, it is shown that the aluminum metal layer will undergo strong deformation when it breaks. The aluminum layers with good plasticity will absorb energy and be deformed under the action of force, and will play an important role in the improving the bending performance of the AlMgTi-based MIL composites.

## 4. Conclusions

This study prepared the AlMgTi-based ternary MIL composites through dual-step hot-pressing method. The high strength and high hardness intermetallic compounds (Mg_17_Al_12_, Mg_2_Al_3_ and Al_3_Ti) and ductile metal layers were found. The transfer layers between the Al/Mg layers decelerate the change in hardness gradient between the ductile and brittle layers, and reduce the stress concentration at the interface effectively. The bending strength of the material can reach 265 MPa, and specific strength is 105 × 10^3^ Nm/kg, higher than the AlTi-based MIL composite. The diffusion layer shows brittle crack morphology, the aluminum layer shows ductile crack morphology, and the magnesium layer shows cleavage fracture characteristics.

## Figures and Tables

**Table 1:** Chemical composition of foil raw materials.

Materials	Chemical Composition (wt.%)
Titanium TA2	Ti:99.91, Cr:0.04, Fe:0.04, Ni:0.01
Aluminum	Al:98.39, Fe:0.50, Cl:0.42, Na:0.25, K:0.15, Si:0.12 Mn:0.04, Cr:0.04, Cu:0.03, Ca:0.03, Ti:0.03
AZ31 alloy	Mg:95.53, Al:3.31, Si:0.04, Mn:0.45, Zn:0.67

**Table 2:** Density and specific strength of AlMgTi-based and AlTi-based MIL composites

Samples	Density (g/cm^3^)	Specific Strength (×10^3^ Nm/kg)
AlMgTi-based MIL composite	2.51	105
AlTi-based MIL composite	3.82	97
